# Food marketing on digital platforms: what do teens see?

**DOI:** 10.1017/S1368980024000235

**Published:** 2024-01-25

**Authors:** Charlene D Elliott, Emily Truman

**Affiliations:** University of Calgary, Communication, Media and Film, 2500 University Drive NW, Calgary, AB, T2N 1N4, Canada

**Keywords:** Teenagers, Food marketing, Persuasive power, Digital media, Marketing appeals, Digital platforms

## Abstract

**Objective:**

Given the aggressive marketing of foods and beverages to teenagers on digital platforms, and the paucity of research documenting teen engagement with food marketing and its persuasive content, the objective of this study is to examine what teenagers see as teen-targeted food marketing on four popular digital platforms and to provide insight into the persuasive power of that marketing.

**Design:**

This is an exploratory, participatory research study, in which teenagers used a special mobile app to capture all teen-targeted food and beverage marketing they saw on digital media for 7 d. For each ad, participants identified the brand, product and specific appeals that made it teen-targeted, as well as the platform on which it was found.

**Setting:**

Online (digital media) with teenagers in Canada.

**Participants:**

Two hundred and seventy-eight teenagers, aged 13–17 years, were participated. Most participants were girls (63 %) and older teenagers (58 % aged 16–17 years).

**Results:**

Participants captured 1392 teen-targeted food advertisements from Instagram, Snapchat, TikTok and YouTube. The greatest number of food marketing examples came from Instagram (46 %) (with no difference across genders or age), while beverages (28·7 %), fast food (25·1 %) and candy/chocolate were the top categories advertised. When it comes to persuasive power, visual style was the top choice across all platforms and participants, with other top techniques (special offer, theme and humour), ranking differently, depending on age, gender and platform.

**Conclusions:**

This study provides insight into the nature of digital food marketing and its persuasive power for teenagers, highlighting considerations of selection and salience when it comes to examining food marketing and monitoring.

Food marketing to teenagers has received considerable attention in recent years, given teens’ unique vulnerability to such marketing^(1,2)^ and its negative impact on food intake^(3)^. Studies have documented the aggressive nature of food marketing to teenagers more generally, pointing to a food environment saturated with messages for products high in fat, salt and sugar^(3–5)^. Yet it is the major shift to digital food marketing that has sparked particular concern, given teenagers’ immersion in digital culture^(6)^. As the US Center for Digital Democracy observed, ‘[f]ood and beverage companies have made digital media ground zero for their youth promotion efforts’^(6)^ ^(p.3)^, weaving unhealthy brands and products throughout the ‘media and cultural experiences that dominate the lives of young people’^(6)^ ^(p.7)^.

While the long-term impacts of such marketing have yet to be seen, the research we do have paints a sobering picture. Content analyses show that teens are exposed to digital promotions for unhealthy foods on Instagram, TikTok, YouTube, Snapchat, Twitter, Facebook, Tumblr and Vine^(7–10)^. Surveys reveal teenagers’ strong engagement with food brands on Facebook and YouTube^(11)^ and also suggest that unhealthy dietary intake may increase as social media engagement increases^(12,13)^. Experimental studies document teens’ greater recall of unhealthy food brands (compared with healthy food or non-food advertising) on Facebook^(14)^ and preference for the visual presentation of Instagram food ads over traditional ads^(15)^. Finally, participatory research with teens reveals the scope and variety of digital food messaging (content and platform wise) targeting teenagers, alongside the appeals that they find salient^(16,17)^.

Despite this recent spate of literature on teenagers and food in the digital marketplace, a number of research gaps exist. Studies that compare data across digital platforms are rare, and little is known about how, or if, targeted food marketing differs across the platforms popular with teenagers. While experimental studies^(14)^ and survey research (often recall studies) seek to capture the impact of food marketing on teenage populations^(12,18)^, few studies engage with *teenagers’ real-life encounters* with food marketing messages and *teens’ own assessment* of the persuasive content or ‘power’ within those messages. Power, here, refers to the content and design elements within an advertisement that capture attention and make it persuasive to consumers^(1,2,19)^. These techniques include humour, celebrity, colour, music and visual style (among others) and are highly targeted in terms of appeal – certain techniques will persuade more than others, depending on the audience. Despite the critical importance of (food) marketing power, understanding of the specific techniques that resonate with a teenage audience is still developing. Examinations of differences by age and gender within the teen audience are rare.

Persuasive power, then, is a critical piece of the puzzle when it comes to food marketing to teenagers. Exposure to food marketing is a second (more documented) piece of that puzzle. Exposure refers to a message’s reach, frequency and impact^(20)^ ^(p.8)^. According to the food advertising hierarchy of effects framework^(21)^, exposure – especially repeated exposure – initiates a domino effect that prompts product recognition, positive attitudes towards the advertised product, and ultimately, the intent to purchase or consume^(21)^. However, given the proliferation of media platforms, the expansion of media use and the endless volume of commercial messages available, a significant quantity of digital advertising might not be noticed or heeded. Although teenagers may be *exposed* to a food advertisement, its mere presence on digital media is not *de facto* evidence of impact. Exposure to advertising does not mean it captures their attention and it does not mean that advertisement is persuasive. This reality is certainly recognised by digital marketers, who flag consumer inattentiveness to advertising as a ‘pesky byproduct’ of the current landscape of infinite content^(22)^ and provide tips on how to ‘stop the scroll’^(22–24)^. Exposure to advertising, in short, is not enough. Consumers must engage with the content and find it persuasive, which makes questions of power all important.

Given this, broad aim of this research is to provide a snapshot of the food marketing messages targeted at teenagers on digital media and to gain insight into what teens find persuasive within that marketing. More specifically, the aims are to understand what teenagers see as teen-targeted food marketing on digital platforms, as well as the persuasive power of that marketing. The question of what teens *see* is understood here in a dual sense, referring both to the food messages teenagers encounter as they go about their (digital) lives and to the specific elements within those food advertisement that they consider to be teen-targeted. Focusing on teens’ real-life encounters with the food marketing that targets them matters because studies estimating exposure^(9,10)^, recall studies^(12,13)^ and experimental studies (in which researchers pre-select examples of food marketing for participants to review)^(14,15)^ do not provide a clear picture of what teenagers *actually see and find persuasive in digital environments.* Importantly, since persuasive appeals are not equally ‘powerful’, asking teens to identify elements of persuasive power *within* those ads provides insight into what is salient to them.

This research is particularly timely in a Canadian context, as it works to inform Health Canada’s M2K (Marketing to Kids) Monitoring strategy focused on food and beverage marketing to young people. The research can also inform more global efforts to monitor adolescent exposure to food marketing by generating insight into (digital) food environments^(25)^. The study provides comprehensive insight into the power of food and beverage marketing to teenagers found on the digital platforms popular with them.

## Methods

Data for this project were drawn from a broader study on food marketing and persuasive power in Canada, in which teenagers (aged 13–17 years) were asked to capture the teen-targeted food marketing they saw in legacy media, digital media and in the built environment for 7 consecutive days and to identify the specific appeals within each ad that made it teen-targeted. To do so, participants used a novel mobile app to capture the ad and to identify its product and brand, as well as the platform and the persuasive appeals within those ads. As noted above, these persuasive appeals constitute the ‘power’ of the advertisement^(19)^. A comprehensive selection of platforms and persuasive appeals was provided within the app: for each advertisement, participants could select the bubble naming the appropriate platform and as many persuasive appeals (or persuasive techniques) as desired. Ten persuasive techniques were provided within the app; these included *visual style, theme, music, celebrity, special offer, humour, teenaged-actor, language (teen specific), interactivity* and *cartoon.* An *other* category was also included, which allowed participants to add additional persuasive appeals (via a free-text field) as necessary. Ethics approval was received for this study (University of Calgary Conjoint Faculties Research Ethics Board, CFREB19-0020; Health Canada Ethics Review Board, REB 2021-020H). Details of app development^(26)^, pilot testing^(16)^ and data cleaning for study have been provided elsewhere^(27)^.

In the broader study, a final sample of 309 participants were recruited from September 2021 to September 2022 from schools, community groups, sports teams and through Instagram. Interested teenagers were first directed to a secure website, which contained detailed information about the study and the consent form. (Given the low-risk nature of the study, and their decision-making capacity as 13–17-year-olds, teenagers were able to provide their own informed consent on this secure website. Doing so generated a unique numerical code that gave the participating teen access to download and use the app.)

The final sample of 309 participants submitted 1825 advertisements for analysis and tagged nineteen platforms on which they found teen-targeted food marketing. While the study provided a comprehensive profile of brands, products and appeals across all platforms, results revealed that digital platforms dominated the ads collected. As such, the present study isolates the most popular platforms captured – Instagram, Snapchat, TikTok and YouTube – for further analysis. Over three-quarters (76·3 %) of the submitted advertisements were found on these four platforms, making them worthy of detailed examination.

After separating the dataset containing ads from Instagram, Snapchat, TikTok and YouTube from the larger study, frequencies and percentages were used to describe the number of advertisements identified on each platform, overall and by participant. Two-sample tests for equality of proportions with continuity correction were used to compare frequencies of platforms identified between genders. For each platform, the food brands and indicators most frequently identified in advertisements by participants were reported using frequencies and percentages. Data analysis was conducted using R version 4.1.2.

## Results

Two hundred and seventy-eight teenagers submitted at least one food one advertisement from Instagram, Snapchat, TikTok or YouTube over the 7-d data collection period. The majority of participants were girls (63 %) and older teenagers (58 % aged 16–17 years). See Table 1. Overall, these participants submitted and tagged 1392 teen-targeted food advertisements.

**Table 1:** Participants reporting advertisements from Instagram, Snapchat, TikTok or YouTube: breakdown by age and gender (*n* 278)

Characteristic	Frequency (*n*)	Proportion (%) (rounded)
Gender		
Girl	176	63
Boy	83	30
Gender non-conforming	19	7
Age		
13	24	7
14	39	14
15	54	19
16	82	30
17	79	28

### Food marketing on digital platforms: considerations by platform and gender

When it comes to food marketing by platform, the greatest number of food marketing examples came from Instagram (*n* 643, 46·2 %), followed by Snapchat (*n* 303; 21·8 %) and TikTok (*n* 235; 16·9 %). Food marketing captured from YouTube comprised 15·2 % of the sample (*n* 211). Table 2 details the number of food advertisements captured by platform and gender, revealing that for the most frequently reported platform, Instagram, no difference exist across genders (*P* = 0·99). However, Snapchat and TikTok were more likely to be reported by girls compared with boys and gender non-conforming participants (39·8 % *v*. 21·6 %, *P* = 0·003; 34·7 % *v*. 21·6 %, *P* = 0·03; respectively). More boys and gender non-conforming participants identified food advertisements on YouTube compared with girls, although this difference was not statistically significant (31·4 % *v*. 21·6 %; *P* = 0·10).

**Table 2:** Frequencies and proportions of participants reporting at least one advertisement, by platform and gender* (*n* 278)

	Girl (*n* 176)		Boy (*n* 83)		Gender non-conforming (*n* 19)	
Platform	*n*	%	*n*	%	*n*	%
Instagram	120	68·2	56	67·5	13	68·4
Snapchat	70	39·8	18	21·7	4	21·1
TikTok	61	34·7	18	21·7	4	21·1
YouTube	38	21·6	26	31·3	6	31·6

*n*, frequency; %, proportion.

* Participants could report advertisements from multiple platforms.

A similar pattern can be seen when the teenagers are analysed by age, insomuch as Instagram was reported by the greatest proportion of participants across all ages: no difference existed comparing younger (aged 13–14 years) and older (aged 15–17 years) participants (*P* = 0·68). Although older teens captured food ads from YouTube more frequently than the younger participants, the difference was not statistically significant (27·0 % *v*. 19·0 %, *P* = 0·27). See Table 3.

**Table 3:** Frequencies and proportions of participants reporting at least one advertisement, by platform and age* (*n* 278)

	13 (*n* 24)		14 (*n* 39)		15 (*n* 54)		16 (*n* 82)		17 (*n* 79)	
Platform	*n*	%	*n*	%	*n*	%	*n*	%	*n*	%
Instagram	16	66·7	25	64·1	35	64·8	58	70·7	55	69·6
Snapchat	7	29·2	12	30·8	13	24·1	31	37·8	29	36·7
TikTok	8	33·3	11	28·2	17	31·5	28	34·1	19	24·1
YouTube	5	20·8	7	17·9	18	33·3	21	25·6	19	24·1

*n*, frequency; %, proportion.

* Participants could report advertisements from multiple platforms.

### Digital platforms: Top food brands and products captured

In the broader study where teenagers captured food marketing across all platforms, coffee and fast-food brands were the most common, with Starbucks, Tim Hortons, McDonald’s and Subway representing the top brands in terms of number of advertisements. Ads for these brands comprised more than one of every five images (22·5 % of the sample). Yet when the food marketing captured by teenagers was examined solely in light of the digital platforms of Instagram, Snapchat, TikTok and YouTube, subtle differences could be seen with respect to ranking (see Table 4). Like the broader study, coffee brands such as Starbucks and Tim Hortons topped the number of ads captured on Snapchat, TikTok and YouTube (with Starbucks promotions comprising 20 % of the Snapchat ads submitted). But with Instagram – the digital platform from which 46·2 % of the ads were reported – the top two brands were not coffee brands, but rather gummy candies (Trolli) and fruit drinks (Fruité). This suggests different targeting strategies by platform, at least for the teenagers consulted. Regardless, the top ten brands captured across these platforms are generally multinational fast food or coffee chains and confectionary products, while the top food and beverage *categories* advertised in these ads are for beverage (28·7 %), fast food (25·1 %) and candy/chocolate (19·0 %) (see Table 5).

**Table 4:** Frequencies and proportions of the top ten food or beverage brands for Instagram, Snapchat, TikTok and YouTube

Instagram (*n* 643)			Snapchat (*n* 303)			TikTok (*n* 235)			YouTube (*n* 211)		
Brand	*n*	%	Brand	*n*	%	Brand	*n*	%	Brand	*n*	%
Trolli	45	7·0	Starbucks	59	19·5	Tim Hortons	19	8·1	Starbucks	17	8·1
Fruité	29	4·5	Jolly Rancher	50	16·5	McDonald’s	15	6·4	Tim Hortons	13	6·2
Tim Hortons	28	4·4	Subway	49	16·2	Circle K	15	6·4	Maynards	12	5·7
McDonald’s	22	3·4	Coca-Cola	45	14·9	Starbucks	12	5·1	Reese’s	11	5·2
Wendy’s	14	2·2	Trolli	17	5·6	Happy Planet	11	4·7	Skip The Dishes	10	4·7
Bobba	14	2·2	Takis	14	4·6	A&W	10	4·3	A&W	10	4·7
Starbucks	13	2·0	McDonald’s	12	4·0	Subway	8	3·4	Dairy Queen	9	4·3
KFC	12	1·9	Tim Hortons	8	2·6	Fruité	8	3·4	Kellogg’s	8	3·8
Dairy Queen	12	1·9	Jelly Belly	6	2·0	Takis	7	3·0	Uber Eats	6	2·8
Hershey’s	11	1·7	Mentos	4	1·3	Jolly Rancher	7	3·0	Hershey’s	6	2·8

**Table 5:** Frequencies and proportions of the top ten food or beverage categories for Instagram, Snapchat, TikTok and YouTube (*n* 1392)

Rank	Category	Frequency (*n*)	Proportion (%)
1	Beverage	399	28·7
2	Fast food	350	25·1
3	Candy/chocolate	265	19·0
4	Snacks	99	7·1
5	Dairy	46	3·3
6	Food delivery app	43	3·1
7	Restaurant	36	2·6
8	Other	36	2·6
9	Refrigerated/frozen food	31	2·2
10	Breakfast food	24	1·7

### Persuasive power and digital platforms

When it comes to persuasive power (the techniques within an advertisement that work to persuade), clear trends were apparent. *Visual style* was the top technique tagged across all digital platforms and selected in similar proportions for Instagram (27·3 %), Snapchat (27·2 %), TikTok (23·7 %) and YouTube (27·0 %). *Visual style* refers to elements within an advertisement (such as colour, font and animated effects) that create its overall feel or effect. *Special offer,* covering limited time products, limited edition products, discounts and combo deals, was also tagged in relatively consistent proportions across the four digital platforms, ranging from 10·2 % (Snapchat) to 15·4 % (Instagram). In fact, the participants’ most selected persuasive techniques did not vary much by platform, with combinations of at least three of the techniques of visual style, special offer, theme (i.e. an overarching idea that pervades the advertisement, such as sports, holidays/seasonal and technology) or humour comprising the top four indicators selected in ads captured on each platform (see Table 6). Certain advertising techniques differed by frequency on each platform, however, reinforcing distinctions between them. For instance, food ads tagged for ‘interactivity’ were far more frequently identified on Snapchat than the other digital platforms, whereas ‘music’ was frequently identified on TikTok and YouTube. Food ads tagged with ‘celebrity’ were far more common on Instagram and TikTok than YouTube and Snapchat (Table 6).

**Table 6:** Frequency of power indicators selected by platform

Instagram (*n* 1224)			Snapchat (*n* 654)			TikTok (*n* 540)			YouTube (*n* 429)		
Indicator	*n*	%	Indicator	*n*	%	Indicator	*n*	%	Indicator	*n*	%
Visual style	334	27·3	Visual style	178	27·2	Visual style	128	23·7	Visual style	116	27·0
Special offer	185	15·1	Interactivity	94	14·4	Music	69	12·8	Music	69	16·1
Theme	168	13·7	Theme	79	12·1	Humour	68	12·6	Special offer	62	14·5
Humour	124	10·1	Humour	75	11·5	Theme	66	12·2	Theme	49	11·4
Celebrity	90	7·4	Special offer	67	10·2	Special offer	56	10·4	Teenaged actor	39	9·1
Cartoon character	73	6·0	Cartoon character	50	7·6	Teenaged actor	42	7·8	Humour	39	9·1
Language	71	5·8	Teenaged actor	39	6·0	Celebrity	38	7·0	Celebrity	23	5·4
Interactivity	64	5·2	Music	34	5·2	Interactivity	36	6·7	Language	12	2·8
Music	63	5·1	Language	19	2·9	Language	27	5·0	Cartoon character	12	2·8
Teenaged actor	49	4·0	Celebrity	17	2·6	Cartoon character	9	1·7	Interactivity	7	1·6

*n*, frequency; %, proportion.

When the persuasive techniques identified in the food advertisements from Instagram, Snapchat, TikTok and YouTube were examined by participant gender and age, results proved fairly consistent. Techniques like visual style, interactivity or theme, etc., that participants tagged in individual food ads captured from SnapChat or TikTok or YouTube or Instagram were similar for the younger and older teen participants and also across genders. However, across all platforms, younger respondents (aged 13–14 years) were more likely to tag advertisements with ‘teenage actor’ than older respondents (aged 15+ years) (8·4 % *v*. 5·4 %, *p* = 0·007). Additionally, in food advertisements collected from Instagram, the youngest teenagers (13 years) were more likely to tag ‘interactivity’ in advertisements (10·6 % *v*. 4·7 %, *P* = 0·01). Boys were also more likely use the ‘humor’ tag on food ads from Instagram compared with girls (12·7 % *v*. 8·3 %, *P* = 0·01).

Elements of creative content are not equally powerful within an advertisement, and different techniques resonate with different audiences. As such, while an advertisement might contain multiple persuasive techniques, participants may only see certain ones as meaningful. For the examples collected and tagged by the teenagers, what cued *teen-targeted* food advertising to them was predominantly visual style, special offer, theme and humour. Beyond this, in 37·4 % of ads teenagers selected only one technique to identify that ad as teen-targeted (see Table 7). Visual style was the single technique selected in one-quarter of the cases (25·7 %), followed by special offer (21·3 %) and celebrity (12·1 %). These single indicators of teen-targeted food marketing should not be glossed over, as they suggest an important insight into persuasive power for this audience, namely, that persuasive power and teen-targeted advertising can be straightforward and *easily identified by teens with a single cue*. For teens, it is not a complex process: they know it when they see it and it does not require multiple indicators to identify.

**Table 7:** Persuasive techniques selected among advertisements where only one technique was selected (*n* 521)

Indicator	Number of advertisements (*n*)	Proportion (%)
Visual style	134	25·7
Special offer	111	21·3
Celebrity	63	12·1
Theme	51	9·8
Humour	48	9·2
Interactivity	38	7·3
Teenaged actor	26	5
Language	21	4
Cartoon character	14	2·7
Music	10	1·9
Other	5	1

## Discussion

Digital platforms have, in many respects, upended food and beverage marketing strategies. They have enabled unprecedented access to young consumers by food companies and brands, big and small, accompanied by precise targeting practices. According to a leading global digital agency, social media ranks as the ‘top food and beverage marketing’ strategy for companies^(28)^ and is a lucrative channel for the food and beverage sector’ considering costs *v*. results^(28)^. Given the popularity of digital platforms with young people and the unhealthy nature of food marketing, it is not surprising that digital food marketing to young people has been deemed ‘a substantial public health problem’^(29)^.

This study sheds light on the nature of that problem. By asking teenagers to capture and tag the food marketing they deem teen-targeted (in terms of platform, product, brand and persuasive appeals), the study provides insight into the digital food marketplace as viewed by the participants. For them, Instagram is the digital platform with the most teen-targeted food marketing, delivering close to half of the advertisements captured – a popularity that did not change regardless of participant age or gender. Participants also captured hundreds of food advertisements from Snapchat, TikTok and YouTube, with Snapchat and TikTok more likely to be reported by girls. That Instagram ranks as a top platform for all teen genders has been observed elsewhere^(30)^, as has teen girls’ higher preference for Snapchat (compared with boys) and teen boys’ higher preference for YouTube (compared with girls)^(30)^.

At base, our research findings join the growing list of studies documenting the ubiquity of food posts^(31)^ and/or marketing on digital platforms^(8–10,16)^ and the unhealthy nature of such marketing^(9,10,16,32)^. Advertisements for beverages, fast food and candy/chocolate comprised most of the submissions, with 73 % of ads captured promoting these products. This finding reinforces the empirical research chronicling the primacy of these three categories of foods when it comes to food marketing to teenagers^(4,16,27,28,33)^, as well as qualitative research in which teenagers observe that beverages such as soda and convenience foods characterise ‘teen foods’^(34)^. Study findings also reveal important nuances across platforms in terms of the specific products offered, given that the top products captured for each platform are not the same: Instagram’s top captured product (by frequency) is Trolli gummy candy, whereas ads for either Starbucks or Tim Horton’s were most frequently captured on Snapchat, TikTok or YouTube. Such comparisons of promoted products across platforms are not found in other research examining food posts and/or marketing across multiple media platforms. Of course, the targeted nature of digital marketing (shaped by algorithms, including social media algorithms and ranking systems) means that products captured on each platform by our study participants reflects their specific experiences and cannot be generalised to a teen population writ large. However, these differences draw attention to the fact that digital media experiences, including products seen, are not identical across platforms.

The study also provides unique insight into the nature of power, arising from a creative methodological approach that does not assume that all persuasive techniques found within an advertisement hold equal weight or sway. One challenge with much of the research on food marketing to young people is that when it comes to documenting ‘power’ within a persuasive message *all techniques are typically treated as if equally salient and equally significant*. Whether for monitoring purposes or a research study, researchers often examine marketing content for the presence of a list of indicators. Similarly, content analyses of child-targeted food marketing tend to capture all appeals (such as the presence of a cartoon character, celebrity, appeal to fun, etc.) that have been determined to be child-directed. Coders then tally up the total number for each and draw conclusions about what it means. However, simply documenting the presence of techniques ‘directed’ at children or teenagers does not mean those techniques are salient to them. When all techniques are captured, there is no means of discerning which ones are more (or less) meaningful. Here, Entman’s^(35)^ cautionary note about content analysis when it comes to framing – a theory used in the field of communication – proves helpful, because it applies equally to the treatment of marketing power. Entman observes that coders who ‘neglect to measure the salience of elements in [a] text’… ‘may often yield data that misrepresent the media messages that most audience members are actually picking up’^(35)^ ^(p.57)^. Heeding this, our study is designed to capture salience and to represent precisely what the teen audience is ‘actually picking up’ from the food marketing that they see. Unlike other research studies that select techniques ‘likely to be popular’ with teenagers^(14)^, participants in our study both selected the food advertisements that they considered to be teen-targeted and the specific techniques within the ad that made it so. In this way, the participating teenagers communicate the salience of particular elements within a food ad, providing insight into marketing power.

Accordingly, we argue that the concepts of selection and salience are essential to understanding food marketing, especially on digital platforms. We do not use these key terms from framing theory precisely as defined in that context (given framing’s general concern with news coverage). However, we do align with framing theory’s aim to understand ‘the power of a communicating text’^(35)^ ^(p.51)^ – and, in this case, we are examining the power of food marketing communications, whereby *selection* speaks to the issue of exposure and *salience* allows for a better recognition of hierarchy and nuance when it comes to power. What the teenagers report as salient within food marketing is, overwhelmingly, visual style. Other salient persuasive techniques are special offer, theme and humour (ranked differently, depending on the platform).

The importance of visual style to teenagers as a technique of persuasive power in food marketing has been observed in other research studies^(16,36)^. Among them, qualitative research has found that teenagers identify the ‘characteristics of the images’ followed by colours as the ‘most memorable’ aspects of food marketing on digital platforms^(37)^ ^(p.5)^ or note teens’ appreciation for food ads with ‘high visual quality’^(31)^. Recent studies examining the visual techniques found within food marketing collected by teenagers also report that ‘bold focus’ (a style characterised by vivid colourful backgrounds and the food product as a central object of the image) dominates this visual style^(38)^. Along with bold focus, ‘bespoke’ is another popular subset of visual style typifying food marketing on digital platforms: the bespoke is characterised by crisp, clean designs, and a focus on food staging and styling^(38)^. Overall, visual style – which our study participants tagged as the top indicator of teen-targeted food marketing across all digital platforms – is a key element of persuasive power and worth further exploration. Its primacy as a marketing technique is reinforced by marketers themselves, who emphasise visual elements and ‘bold, high contrast colors’ as a tool to ‘stop the scroll’^(24)^ or embrace a brand ‘visual identity’ overhaul, one ‘tailored to recognize the demands of an increasingly digital and “phygital” era’, as Pepsi has done^(39)^.

Beyond visual style, other selected techniques are interesting insomuch as they reveal trends in power and targeting strategies across age and/or gender. For example, the youngest teenagers were more likely than to tag food advertisements with ‘teenage actor’ than older teens (across all platforms) and to tag ‘interactivity’ on Instagram food ads. Boys were more likely to tag humour in a food ad – a tendency that has been observed in other studies^(16,36)^. Persuasive techniques selected in the food ads may also reflect important distinctions when it comes to the functionality of the platform. For example, Snapchat filters are interactive by nature, while music is central to TikTok (given the importance of audio memes to the platform). Additionally, celebrity/influencer promotions are historically commonplace on Instagram and growing on TikTok as the platform’s popularity increases. As such, certain elements of power found within food advertising might be tightly bound up with the platform itself.

### Strengths and limitations

This study is interested in food marketing’s persuasive power, as captured and tagged by teenagers themselves. It does not aim to capture correlations between time spent on social media and exposure to food marketing writ large, although this would be a fruitful topic for future studies.

Instead of documenting food marketing power or exposure more generally, the study aims to understand teenager views on teen-targeted marketing and its persuasive appeals. This aim required a unique approach, whereby participants collect the examples of food marketing and identify the specific persuasive techniques within them. That teenagers tag the persuasive techniques is a clear strength, because it sidesteps the challenge generated when (adult) researchers determine and/or speculate on what is persuasive to teenagers within the content of each ad. The study also considers differences by platform and by age and gender, meaning that the teen audience is properly recognised as teen *audiences*. Beyond this, the study introduces the important concepts of selection and salience when it comes to understanding food marketing power and exposure, with salience providing a means both to recognise that techniques of persuasive power are not equally persuasive and to reinforce that they should be considered as nuanced elements that often exist in a hierarchy – where some may be techniques may be extremely persuasive and other factor not at all. Finally, the findings can be considered in light of the WHO’s 2018 report on food marketing that observed how food marketing policies related to ‘pre-digital media only’ and ‘to younger children and not to adolescents’ are ‘markedly insufficient to address the continuing challenges in this field’^(2)^. Teenagers, as the WHO observes, are ‘particularly susceptible’ to unhealthy food marketing for several reasons. First, although teenagers may be more cognitively advanced than children, marketing – and especially digital marketing – has emotional effects that bypass cognitive processing^(2)^. Beyond this, teenagers may not care or wish to resist such marketing^(2)^, particularly given the social currency it has with this audience^(34)^. Insight into the specific persuasive techniques and their salience allows us to consider what this currency entails and why teenagers may lack interest in ‘resisting’ food marketing.

### Conclusion

This study focuses on teen engagement, power and digital platforms, with the goal of illuminating food marketing and its persuasive power as identified by teenagers in light of the most popular digital platforms they use. By selecting the food marketing they deem as teen-targeted and tagging the persuasive techniques salient within those ads, participants not only reveal the food brands and products targeting them but also what they value when it comes to persuasive power across the platforms of Instagram, Snapchat, TikTok and YouTube.
